# Spatiotemporal impact of urban development on nighttime light intensity and its hotspot distribution

**DOI:** 10.1371/journal.pone.0325696

**Published:** 2025-06-11

**Authors:** Tzu-Cheng Chang, Jia-Hong Tang, Ta-Chien Chan

**Affiliations:** 1 Department of Geography, National Taiwan Normal University, Taipei, Taiwan; 2 Research Center for Humanities and Social Sciences, Academia Sinica, Taipei, Taiwan; 3 Institute of Public Health, School of Medicine, National Yang Ming Chiao Tung University, Taipei, Taiwan; 4 Department of Public Health, College of Public Health, China Medical University, Taichung, Taiwan; 5 School of Medicine, College of Medicine, National Sun Yat-sen University, Kaohsiung, Taiwan; Northeastern University (Shenyang China), CHINA

## Abstract

Nighttime light (NTL) data serve as a valuable proxy for accessing urbanization and socio-economic activities at various scales. This study investigated the spatiotemporal evolution of NTL intensity in Taipei City from January 2018 to June 2023 using data from the Visible Infrared Imaging Radiometer Suite (VIIRS) Day/Night Band (DNB) via the Google Earth Engine (GEE) platform. A grid system comprising 1,211 cells (500-m resolution) was established to integrate land use, road networks, population, electricity consumption, and business prosperity into temporal, spatial, and spatiotemporal models using Integrated Nested Laplace Approximations (INLA). Additionally, spatiotemporal patterns were analyzed through the space–time cube in ArcGIS Pro. This finding highlights the strong influence of commercial activities and electricity consumption on NTL intensity, with persistent hotspots in commercial and industrial areas and cold spots in forested and agricultural zones. This study underscores the potential of NTL data to capture the interplay between urbanization, land use, and socioeconomic factors. Emphasizing land use as a central analytical focus provides a scalable framework for future urban studies and policy development that can be applied to diverse urban contexts.

## Introduction

Urbanization and its intricate interplay with socioeconomic dynamics, land-use changes, and ecological environments present critical challenges for urban planners and policymakers globally. Satellite-derived Nighttime light (NTL) data have emerged as powerful tools for analyzing urbanization trends [1,2], human dynamics [3,4], and socioeconomic [5] and environmental implications [2,6]. The transition from the Operational Line-scan System to the Visible Infrared Imaging Radiometer Suite (VIIRS) Day/Night Band (DNB) has significantly enhanced the spatial and spectral resolution of NTL observations, enabling more precise analysis of urban systems [7,8]. Such advancements have broadened the potential applications of NTL data, making them valuable proxies for assessing urban development and related phenomena [9].

Urban growth is not a uni‑dimensional process; it unfolds through coupled changes in land‑use allocation, socio‑economic restructuring, and ecological feedback. Recent studies on heat‑vulnerability optimization in climate‑adaptive cities [10], Markov‑FLUS–based simulations of land‑use change and ecosystem‑service valuation in Ezhou [11], and community heat‑risk variations in Shenyang [12] collectively show that accelerating urbanization can exacerbate thermal inequality and degrade ecosystem services when governance is weak. Evidence from the middle reaches of the Yangtze River agglomerations further indicates that human–air–ground interactions amplify local warming and pollutant accumulation under rapid land‑conversion trajectories. Likewise, attribution studies of urban thermal‑environment change reveal that anthropogenic exploitation, rather than background climate alone, drives most brightness‑linked heat signatures in mainland Chinese megacities [13]. Outside China, micro‑climatic experiments in Burdur, Türkiye, confirm that surface materials and built‑form typologies modulate both thermal comfort and night‑time luminance [14]. These findings converge on a key insight: urban land‑use mix and human activity intensity jointly reshape nocturnal light emissions and environmental outcomes, underscoring the need to integrate fine‑resolution NTL with multi‑source land‑use and socio‑ecological data, as pursued in the present study.

Recent studies have increasingly employed NTL data to explore diverse socioeconomic phenomena such as urban growth [15,16], economic development [17], and environmental impacts [18]. Fine-scale NTL studies have demonstrated pronounced links between land-use composition and urban brightness [19–22]. However, most of these studies either analyzed a single snapshot in time, did not consider Bayesian spatiotemporal inference, or focused on cities with relatively low population densities. Compact Asian megacities, such as Taipei, might understate vertical land-use intensity and mixed-use lighting signatures, thus misguiding land-use or energy policies. Moreover, they relied on one-off or annual composites, ignoring short-term shocks and seasonal lighting cycles. This can overestimate long-term growth trends or mask temporary downturns, leading planners to misestimate the pace of urban expansion. Urban land-use patterns, characterized by residential, commercial, industrial, and mixed-use zones, play a crucial role in shaping NTL intensity [23]. For example, commercial hubs and high-density residential areas typically exhibit increased brightness, whereas agricultural and forested regions are dimmer [24–26]. By focusing on the link between NTL and land use, this study aimed to uncover patterns that transcend geographic boundaries, providing broader applicability and generalizability.

Land use is a critical lens through which the dynamics of urbanization can be understood. Unlike analyses confined to specific regions, framing NTL studies within the context of land use allows meaningful cross-regional comparisons. Urbanization alters land use patterns, which in turn influences the spatial distribution and intensity of NTLs. Recent studies demonstrated that commercial areas and mixed-use zones are strongly correlated with increased NTL intensity, whereas areas dominated by agriculture, forestry, and public facilities typically exhibit weaker light emissions [27,28]. However, understanding these relationships requires a methodological framework capable of capturing the complex spatiotemporal interactions between NTL intensity and land use type [29]. The widespread use of simple OLS or spatial lag models often neglects spatiotemporal autocorrelation. Omitted correlations may misattribute brightness changes to socioeconomic drivers instead of latent spatial processes. These shortcomings highlight the need for a fine-grained Bayesian spatiotemporal framework that integrates land-use heterogeneity, which is the approach adopted in this study. By explicitly addressing snapshots, scales, and model biases, our work offers reliable evidence for energy-efficient urban planning and cross-regional comparability.

This study adopts land use as its central theme, aiming to provide insights that are relevant to Taipei City and are transferable to other urban contexts. By integrating land use data into a spatiotemporal modeling framework, we aim to demonstrate how urbanization processes shape light emissions and how NTL can serve as a robust indicator of socioeconomic activity across diverse geographic regions.

This study seeks to advance the understanding of how urbanization and socioeconomic activities shape NTL intensity by integrating fine-grained land use data into a spatiotemporal Bayesian framework. The objectives of this study are as follows:

1. Analyze the influence of different land use types on NTL intensity.
2. Examine the spatiotemporal dynamics of NTL in the context of urban expansion and economic activity.
3. Validate the applicability of the INLA approach for large-scale spatiotemporal NTL analysis, thereby setting a methodological precedent for future research.

By emphasizing land use as the focal point, this study not only addresses and extends existing gaps in NTL research but also provides transferable insights that can inform urban planning and policy in diverse settings.

## Materials and methods

### Research area

With a history of over a hundred years, Taipei has become Taiwan’s political, economic, and cultural center, disseminating comprehensive charm everywhere. It is one of the few rare cities that possess a unique set of natural characteristics, including mountains, forests, rivers, plains, wetlands, and farmland. It offers a unique urban environment with high population density, diverse land use, and rapid development. With over 2.5 million people in just 271.8 km^2^, it is ideal case to study the relationship between NTL intensity and socioeconomic activity. Its mix of commercial, residential, industrial, and green spaces highlights spatial and temporal NTL variations [30,31]. Similar to cities such as Tokyo, Seoul, and Hong Kong [32,33], Taipei faces challenges in energy use, urban sprawl, and sustainability, making comparative studies valuable. Its urban planning policies focus on energy efficiency and sustainable development, further supporting NTL as a proxy for economic activity [34]. Although Taipei’s unique characteristics may limit the generalizability of the findings, the study’s framework emphasizes mechanisms such as the correlation between land use and NTL that are applicable to other cities [34]. Taipei serves as an exemplary case study for exploring the impact of urban planning on socioeconomic dynamics and environmental sustainability [33].

### NTL data

This study utilized NTL data from [35], sourced from the Google Earth Engine [36] platform, which is a comprehensive tool for analyzing and visualizing extensive open-source satellite imagery datasets. The GEE platform simplifies the initial processing of satellite images, circumventing the challenges associated with large file sizes and extensive preprocessing requirements. It offers a range of APIs and tools that facilitate the development of new analytical methods and application models [37].

The dataset employed in this study was VNP46A2, a product of the VIIRS DNB sensor onboard the Suomi National Polar-orbiting Partnership (NPP) satellite. The sensor captures terrestrial visible and near-infrared (NIR) light at night. The VNP46A2 product, also known as the VIIRS/NPP Gap-Filled Lunar Bidirectional Reflectance Distribution Function (BRDF)-Adjusted Nighttime Lights Daily L3 Global 500 m Linear Lat/Lon Grid, offers data corrected for moonlight and atmospheric conditions using a BRDF. With a spatial resolution of approximately 500 m (15 arcseconds), this dataset provides fine-scale temporal resolution, theoretically daily, and passes over Taiwan at 1:30 AM. The dataset extends from January 19, 2012, to the present and is continuously updated. The 16-bit radiance values of the NTL data range from 0 to 6553.4, allowing for precise representation of illumination levels from very dim to very bright sources [9].

For this study, a grid overlay of 500 m cells was created based on the administrative boundaries of Taipei City, totaling 1,211 cells. This grid was uploaded to the GEE for local invocation. The temporal range was from January 2018 to June 2023, spanning 66 months. The preprocessing involved the selection of the appropriate grid, time frame, and bands for download, followed by the averaging of the NTL values within each grid cell to represent the NTL intensity. Because of cloud cover, not all days had valid data; thus, monthly averages were computed from daily values to mitigate this concern.

### Land use and road network data

Land use data were obtained from the National Land Surveying and Mapping Center 2021 land use survey results for Taipei City (https://maps.nlsc.gov.tw/). The land use classification includes three hierarchical levels. The first level has nine categories: agricultural usage, forest, transportation, building including business, residential and mix-residential usage, public access, manufactural factory, and roads. The second and third levels further differentiate from the first level, with the second level having 48 categories and the third level having 93 categories. In this study, we primarily utilized the first and second levels owing to the overly fragmented nature of the third-level data.

The land use types incorporated in this study included commercial, residential, mixed-use, and industrial areas, forests, agricultural lands, and public utilities, and excluded the road-related subclasses from transportation land use. Road data were obtained from Richi Tech’s 2019 nationwide road network dataset, extracted for Taipei City’s scope, and included road length and area. Road networks were extracted to measure infrastructure density and urban connectivity, which are critical predictors of NTL intensity [28].

Preprocessing involved calculating the proportion of each land use type and road area within each grid cell and normalizing the values between 0 and 1. Road lengths were converted to kilometers to ensure consistency across variables.

### Population, electrical, and business indicators

Population data were obtained from the monthly statistics provided by Taipei City’s Civil Affairs Bureau (https://ca.gov.taipei/), covering the population and household numbers of each basic statistical area (BSA) from 2015 to 2024. The population density for each district was calculated and adjusted based on the proportion of the district area within each grid cell and summed to represent the grid cell population in thousands.

Electricity consumption data were sourced from Taiwan Power Company’s public data on county-level electricity usage. The processing mirrored that of the population data, adjusting consumption values based on the proportionate area of each BSA within the grid cells with units of kilowatt-hours (thousands) (https://data.gov.tw/dataset/83422).

Business indicators were developed based on statistical data of business registrations from Taipei City’s Department of Economic Development, covering new establishments, changes, and closures. Geocoding was used to convert the address data to geographical points and compute the monthly variations in business registrations within each BSA. The Min–Max method was used to calculate the economic index, defined as (number of businesses in a month – minimum number of businesses during the study period)/ (maximum number of businesses during the study period – minimum number of businesses during the study period). This index captures the net increase in business activities relative to the maximum and minimum monthly changes during the study period, normalized between 0 and 1, and represents the change in business activities for that month relative to the variations observed throughout the study period (https://www.tcooc.gov.taipei/News.aspx?n=7C9F87E0377FCFBD&sms=20686 https://www.tcooc.gov.taipei/News.aspx?n=536FD32C56F537F3&sms=65021DAA6C6F68CC). Business activity indices were constructed based on monthly registrations and closures [38,39].

### Statistical analysis

#### Bayesian analysis.

The INLA approach was chosen for its ability to handle the complexities of spatiotemporal modeling with high computational efficiency. Unlike traditional Markov Chain Monte Carlo methods, which can be computationally expensive for high-dimensional data, INLA rapidly approximates the posterior distributions, making it ideal for large-scale analyses [40]. The Bayesian framework inherent in INLA accommodates spatial adjacency matrices and autoregressive temporal structures, which are crucial for capturing spatiotemporal heterogeneity in NTL intensity across urban landscapes [41].

A key advantage of INLA is its use of the stochastic partial differential equation (SPDE) approach, which translates continuous spatial processes into a discretized mesh, facilitating the fine-grained estimation of spatial and temporal correlations [42]. This capability is particularly advantageous for analyzing NTL data because it enables the model to capture localized urban phenomena while accounting for broader temporal trends. By integrating land use and socioeconomic variables, the INLA framework provides a robust and flexible methodology for understanding how urbanization and economic activity influence NTL patterns [43].

#### Model specification.

Three types of models were constructed to isolate the temporal, spatial, and spatiotemporal effects.

1. **Temporal Model**

The temporal model uses an autoregressive process of order 1 (AR(1)) to account for temporal autocorrelation in the NTL data. The AR(1) structure assumes that the NTL intensity at a given time point is influenced by its value during the previous month. This approach has been shown to be effective in capturing time-dependent dynamics in urban and economic data [40]. The model is specified as follows:


$$Y_{t}=\;\alpha +\;\beta X_{t}+\;\phi Y_{\left\{t-1\right\}}+\;\epsilon_{t}$$ (1)

where *Y*_*t*_ represents NTL intensity, *ϕ* is the autoregressive parameter, and *∊*_*t*_ is error term. Variables such as electricity sales, population, business index, and other land use ratios were included as covariates, and $X_{t}$ explained the temporal, spatial, and spatiotemporal variations in NTL intensity, reflecting changes in economic activities and infrastructure over time.

1. **Spatial Model**

The spatial model incorporates a Gaussian Random Field with a Matérn covariance function to capture spatial dependencies. Land use proportions and socioeconomic variables were included as covariates. Gaussian Random Fields have been widely used for spatial data modeling because of their flexibility in representing spatial structures [41]. The model can be expressed as follows:


$$Y_{s}=\;\alpha +\;\beta X_{s}+\;f(s)+\;\epsilon_{s}$$ (2)

where s represents grids and f(s) denotes the spatial random effect. Variables such as electricity sales, population, business index, and other land use ratios were included as covariates, and $X_{s}$ explained the spatial variations in NTL intensity, reflecting changes in economic activities and infrastructure over time. The Matérn covariance function was employed in this study due to its robust theoretical foundation and practical advantages in spatial statistics [44]. This function provides exceptional flexibility in modeling spatial dependencies through its smoothness parameter (ν), which allows precise control over the spatial correlation structure. In the context of NTL analysis, where light intensity often exhibits complex spatial patterns due to varying urban structures, the Matérn function’s ability to adapt to different degrees of spatial smoothness is particularly valuable [44]. Furthermore, its implementation within the SPDE approach enables efficient computation for multiple-scale spatial data while maintaining model accuracy [41].

1. **Spatiotemporal Model**

The spatiotemporal model combines the AR(1) and Gaussian Random Field components within a Bayesian hierarchical framework. The SPDE approach maps continuous spatial processes onto a discretized mesh, thereby improving computational efficiency [26,42]. This hybrid model is particularly suited for analyzing dynamic urban phenomena in which temporal and spatial interactions are intertwined. The model is specified as follows:


$$\begin{array}{c} Y_{\left\{s,t\right\}}=\alpha +\beta X_{s,\;t}+f(s)+g(t)+h(s,t)+\epsilon_{\left\{s,t\right\}} \end{array}$$ (3)

where g(t) represents temporal effects and h(s,t) captures spatiotemporal interactions. Variables such as electricity sales, population, business index, and other land use ratios were included as covariates, and $X_{s,\;t}$ explained the spatiotemporal variations in NTL intensity, reflecting changes in economic activities and infrastructure over time.

#### Hotspot analysis.

Using the INLA-estimated NTL values, a spatiotemporal hotspot analysis was conducted using the Space Time Cube tool in ArcGIS Pro. This tool aggregates data into a three-dimensional cube, with the X- and Y-axes representing spatial locations and the Z-axis representing temporal slices. The tool identifies statistically significant clusters of persistent hotspots and cold spots by analyzing the aggregated data, revealing patterns of urban and economic activity [27].

The Emerging Hot Spot Analysis algorithm was employed to track dynamic changes in brightness across Taipei City. Persistent hotspots were commercially identified and correlated with sustained economic activity. Conversely, long-term cold spots were observed in mountainous and undeveloped regions, reflecting a minimal urban influence. This methodology allowed the visualization of temporal trends in NTL intensity, providing actionable insights into urban planning and economic development [45].

## Results

**Fig 1:**
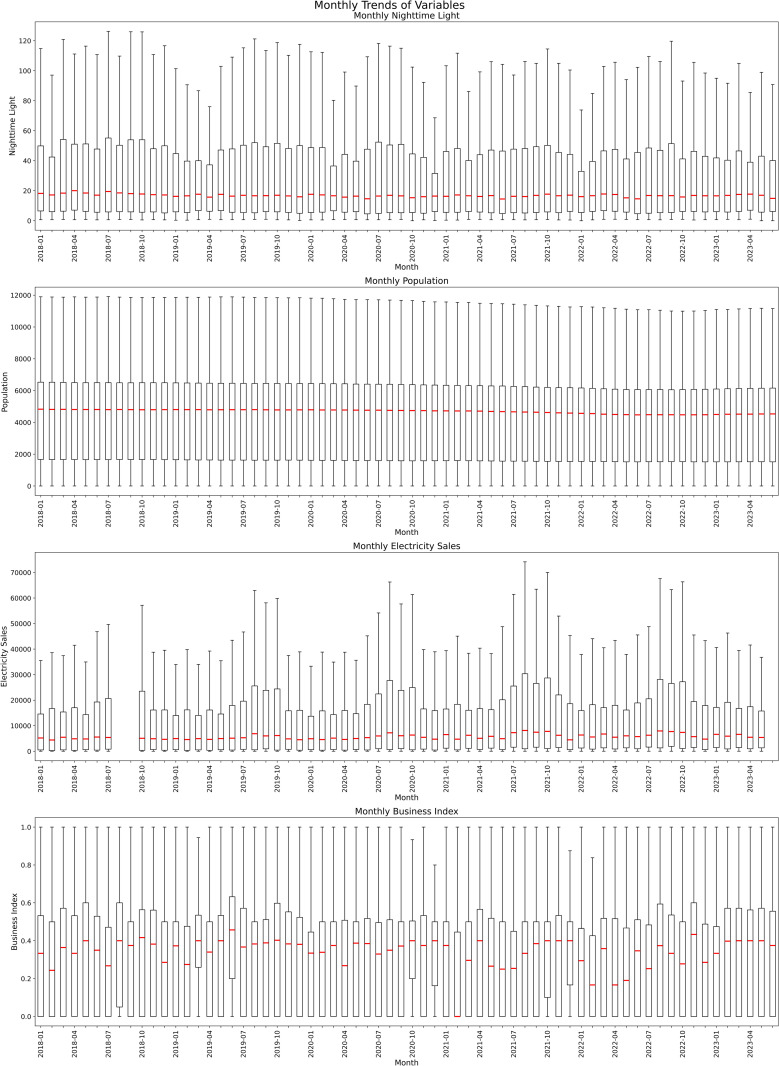
Time series boxplots of: (A) NTL intensity (nW/cm²/sr), (B) Population (k), (C) Electricity Sales (kW), and (D) Business Index (%) from January 2018 to June 2023.

**Fig 2:**
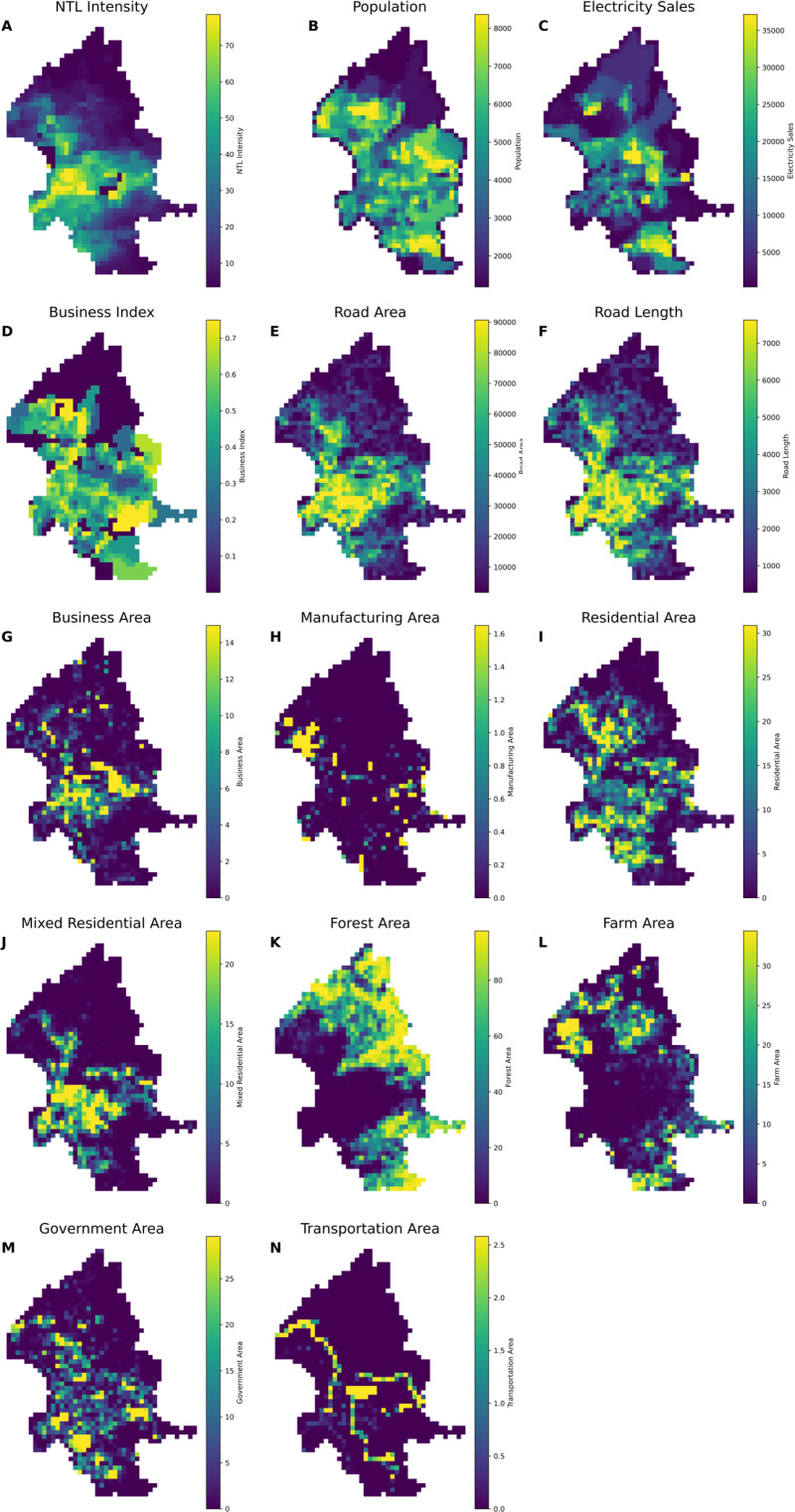
Spatial distributions of the average values across different variables of: (A) NTL intensity (nW/cm²/sr), (B) Population (k), (C) Electricity Sales (kW), and (D) Business Index (%), (E) Road Area (km^2^), (F) Road Length (km), (G) Business Area (%), (H) Manufacturing Area (%), (I) Residential Area (%), (J) Mixed Residential Area (%), (K) Forest Area (%), (L) Farm Area (%), (M) Government Area (%), and (N) Transportation Area (%) in Taipei City. The basemap is republished from Government open data (https://data.gov.tw/dataset/121199) under a CC BY 4.0 license, with permission from Department of Civil Affairs, Taipei City Government, original copyright 2012.

To elucidate the relationship between socioeconomic factors and NTL, we incorporated variables such as population, electricity sales, commercial indicators, roads, and land use into the temporal, spatial, and spatiotemporal models constructed within the INLA framework. This allowed us to obtain the predictive values for each variable. Table 1 presents the descriptive statistics of each variable in the three models. NTL and electricity usage showed wide ranges, indicating that while most areas exhibit low to moderate values, a small number of regions showcase exceptionally high levels. This pattern was similarly reflected in variables such as population and road length, suggesting that only a few areas are densely populated or heavily developed, whereas the majority remain much less developed. Additionally, indicators such as the Business Index and certain land use percentages (including business, manufacturing, and residential categories) often had medians and upper quartiles near zero, implying that these characteristics are concentrated in a limited number of locations. In contrast, some land cover types, most notably forested areas, exhibited a broad spectrum of values of up to 100%, accompanied by high variability. Fig 1 illustrates the monthly average NTL intensity across the study period, revealing stable trends with minor seasonal fluctuations. Figs 1(A) and 1(C) show clear seasonal variations in NTL and electricity sales, with electricity usage peaking during summer months. In Fig 1(B), the population shows a slight decline, and the overall trend remains relatively stable. In contrast, Fig 1(D) shows no clear trend changes over the observation period. In Fig 2 demonstrates the spatial distribution of average values across different variables. High-value clusters are predominantly concentrated in the core areas of Taipei City in Figs 2(A), 2(D), 2(E), 2(F), 2(G), 2(J), and 2(M). In contrast, Figs 2(H), 2(K), and 2(L) display high-value clusters in the peripheral regions of the city. In Fig 2(N), high values are observed along major transportation routes, including the national highway, expressway, and the vicinity of Songshan International Airport, which is centrally located in Taipei City. The remaining figures do not exhibit any distinct spatial distribution patterns.

**Table 1:** Summary statistics for the main variables in our analysis.

	Min	Q1	Median	Q3	Max	Mean	Standard deviation
NTL (nW/cm²/sr)	.000	5.750	16.810	45.360	237.440	27.350	26.010
Population (k)	.000	1.600	4.670	6.290	11.910	4.300	2.620
Electricity (kw)	.000	.790	5.630	18.220	122.180	11.080	13.140
Business Index	.000	.000	.360	.510	1.000	.320	.270
Road Area Ratio (%)	.000	.020	.060	.200	.630	.120	.120
Road Length (km)	.000	.720	1.950	4.680	10.680	2.820	2.5400
Business (%)	.000	.000	.220	2.510	54.580	2.8400	6.170
Manufacture (%)	.000	.000	.040	.130	29.460	.360	1.900
Residential (%)	.000	.230	3.370	13.710	56.280	8.340	10.450
Mix Residential (%)	.000	.000	.020	5.760	46.030	4.330	7.890
Forest (%)	.000	.000	32.720	74.590	100.000	39.090	36.940
Farm (%)	.000	.000	.930	8.800	97.520	7.450	13.470
Government (%)	.000	.000	.560	6.930	100.000	6.270	12.850
Transportation (%)	.000	.000	.020	.080	100.000	.980	6.530

As shown in Table 2, the temporal model highlights the significant role of socioeconomic factors in shaping NTL intensity over time. Population growth (β = 9.955, 95% Credible Interval (2.632, 17.304)) and electricity consumption (β = 0.236, 95% Credible Interval (0.236, 0.41)) emerged as the key drivers of increased brightness. These findings align with urban development trends, in which higher population densities and energy usage reflect intensified urban activities. These results demonstrate that temporal models are effective in capturing long-term changes in urban dynamics but are limited in uncovering localized spatial heterogeneity.

**Table 2:** Posterior estimates (posterior mean, standard deviation (SD) and 95% credible interval) of covariates for the NTL temporal, spatial and spatiotemporal models.

Covariate	Temporal model			Spatial model			Spatiotemporal model		
	Posterior mean	SD	95% Credible Interval	Posterior mean	SD	95% Credible Interval	Posterior mean	SD	95% Credible Interval
Population (k)	19.804	8.521	2.996, 36.494	−0.737	0.743	−2.195, 0.721	0.102	0.724	0.060, 0.144
Electricity (kw)	5.007	2.965	−0.843, 10.819	0.578	0.289	0.011, 1.145	0.032	0.021	0.021, 0.044
Business Index	1.955	30.561	−58.073, 61.781	24.795	4.047	16.857, 32.731	0.987	0.006	0.645, 1.329
Road Area Ratio (%)	0.000	31.623	−62.009, 62.009	0.382	0.031	0.320, 0.443	25.540	0.174	24.461, 26.620
Road Length (km)	0.000	31.623	−62.009, 62.009	−0.810	0.420	−1.633, 0.013	−0.300	0.551	−0.357, −0.243
Business (%)	0.000	31.623	−62.009, 62.009	0.573	0.055	0.466, 0.680	0.169	0.029	0.161, 0.176
Manufacture (%)	0.000	31.623	−62.009, 62.009	0.058	0.149	−0.235, 0.351	−0.008	0.004	−0.028, 0.013
Residential (%)	0.000	31.623	−62.009, 62.009	−0.264	0.041	−0.345, −0.183	−0.029	0.010	−0.035, −0.022
Mix Residential (%)	0.000	31.623	−62.009, 62.009	0.512	0.058	0.398, 0.626	0.129	0.003	0.120, 0.138
Forest (%)	0.000	31.623	−62.009, 62.009	−0.214	0.013	−0.239, −0.189	−0.047	0.005	−0.050, −0.045
Farm (%)	0.000	31.623	−62.009, 62.009	−0.184	0.023	−0.229, −0.139	−0.073	0.001	−0.077, −0.068
Government (%)	0.000	31.623	−62.009, 62.009	−0.018	0.024	−0.065, 0.029	−0.023	0.002	−0.027, −0.019
Transportation (%)	0.000	31.623	−62.009, 62.009	0.224	0.043	0.140, 0.309	−0.062	0.005	−0.071, −0.053
Population * Electricity	−1.065	0.719	−2.475, 0.354	0.004	0.016	−0.028, 0.036	−0.098	0.037	−0.170, −0.026
Population * Business Index	2.914	8.597	−13.939, 19.787	−0.090	1.949	−3.913, 3.734	−0.022	0.007	−0.036, −0.008
Electricity * Business Index	−1.047	2.123	−5.225, 3.125	−0.428	0.788	−1.973, 1.117	−0.002	0.001	−0.003, 0.000

The spatial model underscored the critical influence of land use on NTL intensity (Table 2). Urban regions with higher proportions of commercial zones (β = 21.296, 95% Credible Interval (16.489, 26.103)) and road area ratios (β = 3.121, 95% Credible Interval (65.616, 181.864)) exhibited significantly higher NTL values. These findings highlight the spatial concentration of light emissions in economically vibrant and infrastructurally dense regions. Conversely, regions dominated by residential (β = −1.03, 95% Credible Interval (–0.491, –0.333)), forest (β = −0.229, 95% Credible Interval (–0.255, –0.204)), and agricultural (β = −0.203, 95% Credible Interval (–0.246, –0.154)) land uses showed reduced brightness. These patterns suggest that efficient urban planning and energy-saving measures may limit light emissions in densely populated residential zones, whereas undeveloped land contributes minimally to the NTL intensity.

Finally, the spatiotemporal model integrates the spatial and temporal components, providing a comprehensive view of NTL dynamics influenced by land use. Commercial activity (β = 0.385, 95% Credible Interval (0.201, 0.216)) and mixed-use zones (β = 0.056, 95% Credible Interval (0.188, 0.205)) showed sustained positive effects on brightness, reflecting their role in driving urban lights capes. Forest (β = −0.059, 95% Credible Interval (–0.062, –0.057)) and agricultural (β = −0.088, 95% Credible Interval (–0.092, –0.084)) land uses continued to exhibit negative correlations with NTL intensity, reinforcing their association with minimal urbanization. By leveraging the SPDE framework, the spatiotemporal model was able to capture the evolving brightness patterns in urban districts. For example, commercial hubs in Taipei demonstrated persistent brightness growth, whereas older residential zones exhibited declining NTL intensities. This duality reflects shifts in economic activity and urban policies over time.

The spatial model revealed that land-use composition is a critical determinant of NTL intensity. Areas characterized by higher proportions of commercial land (Business (%): β = 0.709, 95% Credible Interval (0.601, 0.818)) and mixed residential land (β = 0.616, 95% CI (0.502, 0.731)) exhibit significantly elevated brightness levels. In contrast, regions with predominant residential land (β = –0.412, 95% CI (–0.491, –0.333)), forest cover (β = –0.229, 95% CI (–0.255, –0.204)), and agricultural land (β = –0.200, 95% CI (–0.246, –0.154)) are associated with reduced NTL intensity. These findings indicate that economic and mixed-use zones are the primary contributors to urban brightness, whereas natural and predominantly residential areas have moderating influences.

**Fig 3:**
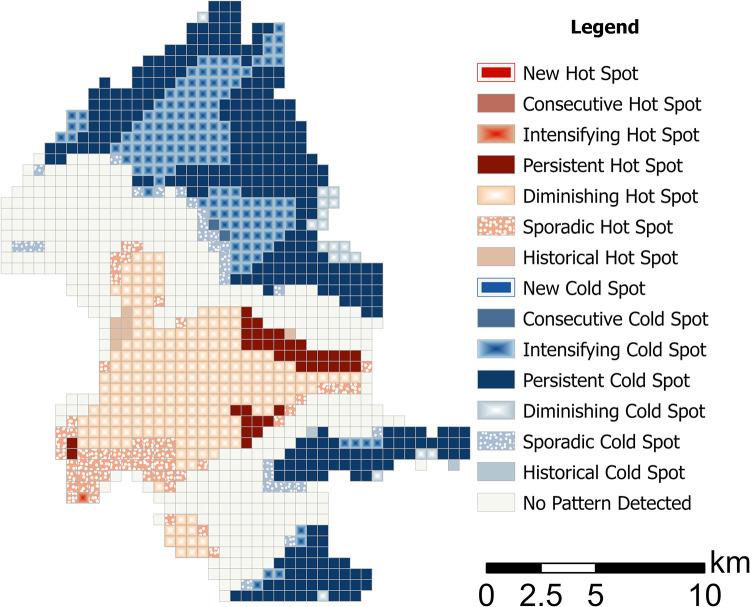
Hot and cold spots in NTL in Taipei City using space–time cube analysis. The basemap is republished from Government open data (https://data.gov.tw/dataset/121199) under a CC BY 4.0 license, with permission from Department of Civil Affairs, Taipei City Government, original copyright 2012. Visualization of predicted NTL values for Taipei City using a 500-m spatial resolution grid, derived from the spatiotemporal INLA model.

Consistent with the spatial findings, commercial activities (as measured by the Business Index and Business (%)) generated sustained high levels of brightness. The space–time cube analysis corroborates the presence of persistent hotspots in Taipei’s central commercial and industrial districts. Peripheral areas and zones dominated by residential and natural land use remained consistently dim. These contrasts underscore the evolving spatial patterns associated with urban expansion and localized economic policies. The significant negative interaction between population and electricity consumption (β = –0.098, 95% CI ( –0.170, –0.026)) and between population and the business index (β = –0.022, 95% CI (–0.036, –0.008)) indicates that as population density increases, the marginal impact of electricity consumption and business activity on NTL intensity diminishes. This suggests that in densely populated areas, additional increases in electricity usage or business activity contribute less to the overall brightness, possibly owing to saturation effects or efficiency improvements. In contrast, the interaction between electricity consumption and the business index (β = –0.002, 95% CI( –0.003, 0.000)) is not statistically significant, implying that the combined effect of these two variables does not deviate from the sum of their independent effects. In essence, although the population moderates the influence of electricity and business activities on NTL, there is no substantial synergistic or antagonistic interplay between electricity consumption and business activity. The space–time cube analysis (Fig 3) reveals clear spatiotemporal dynamics in NTL intensity driven by land use patterns. Persistent hotspots were concentrated in commercial and industrial areas, indicating sustained economic activity and urban infrastructure development. In contrast, long-term cold spots were predominantly located in mountainous and forested regions, reflecting limited urban influence. Emerging trends highlighted significant changes in the central urban districts. For instance, hotspot analysis showed diminishing brightness in residential areas, likely due to shifts in urban policies that prioritize energy efficiency or economic decentralization. These results emphasize the importance of integrating land use data into spatiotemporal frameworks to capture the nuanced interplay between urban development and NTL intensity.

## Discussion

This study highlights the utility of NTL data in capturing the spatiotemporal dynamics of urban development, particularly when integrated with land use data. Commercial activity and electricity consumption are the strongest predictors of NTL intensity, reinforcing the link between economic activity and urban brightness [16,25] The findings also revealed a negative correlation between population density and NTL intensity in the spatial model, suggesting that energy-efficient lighting policies can be implemented in densely populated residential zones. This contrasts with findings from less-developed regions, where a higher population density often corresponds to greater NTL brightness owing to less stringent energy regulations [24].

The space–time cube analysis further demonstrated that land use plays a pivotal role in shaping persistent hotspots, with commercial and industrial zones exhibiting sustained brightness, whereas agricultural and forested areas remain cold spots. These results emphasize the relevance of land use as a critical factor in understanding urban brightness patterns.

By shifting the analytical focus from regional studies to land use-based analyses, this study enhanced the generalizability of the findings. Land use categories, such as commercial and mixed-use zones, consistently showed a positive effect on NTL intensity, reflecting the concentration of human activities in economic hubs. These areas not only emit stronger NTL signals but also act as indicators of urban vitality and economic productivity [28,46].

In contrast, forested and agricultural areas were associated with lower NTL intensities and served as natural cold spots. These findings align with research highlighting the environmental impacts of urban sprawl, as the expansion of urbanized land often encroaches on natural habitats, thereby reducing areas of minimal illumination [15]. These results suggest that NTL data, when paired with land use information, can effectively capture the trade-offs between urban growth and environmental preservation.

The use of INLA combined with the SPDE approach provides a robust framework for spatiotemporal modeling. The computational efficiency and ability of INLA to incorporate spatial adjacency matrices enable a detailed exploration of the relationships between land use and NTL intensity [40,47]. Compared with traditional methods, such as spatial autoregressive models, INLA offers faster and more reliable posterior estimates, even with high-dimensional data [20,41].

Furthermore, the integration of the space–time cube analysis validated the model results by visualizing dynamic changes in urban brightness. Persistent hotspots in economic hubs highlight the enduring impact of concentrated commercial activities, whereas diminishing hotspots in residential zones indicate shifts in urban energy policies and economic activities. These methodological advancements establish a replicable framework for future NTL studies focusing on land use dynamics. Additionally, the establishment of industrial zones, such as the Neihu Technology Park, designated by government planning, contributes to the sustained high brightness in these areas. Conversely, the designation of protected areas or cemeteries in mountainous regions results in a persistently low brightness. The development of commercial centers, such as Taipei 101 in the Xinyi Special District, through urban redevelopment projects has led to long-term increases in NTL intensity.

This study identifies notable discrepancies with the existing literature. For instance, Zhou et al. [16] reported a positive correlation between population density and NTL across China. However, the negative correlation observed in Taipei highlights the influence of localized urban lighting strategies and energy efficiency policies. This suggests that NTL dynamics are context-dependent and influenced by regional governance and socio-economic structures.

Compared to Wu et al. [48], who employed a data-driven approach to measure urban night-time vitality using multi-source urban data, including Meituan data from e-commerce platforms and street-view images, our study differentiated itself by integrating high-resolution NTL remote sensing data with detailed land-use classifications. Whereas Wu et al. [49] emphasized the role of human perception and urban spatial structures in shaping nighttime vitality, our approach provides a more quantitative assessment of urban dynamics through the lens of light emissions, enabling the identification of long-term trends and policy impacts.

Moreover, in contrast to the results of Dong et al. [50], who evaluated the potential for the compound use of urban municipal infrastructure land in Shenzhen using multi-criteria decision-making models and GIS techniques, our study advanced the methodological framework by applying the INLA model for spatiotemporal analysis. This allows for the capture of complex spatial and temporal dependencies in NTL intensity, offering a robust analytical framework that can be generalized to other urban contexts. Dong et al.‘s focus on land use efficiency and infrastructure optimization complements our emphasis on socioeconomic activity indicators, highlighting the multifaceted nature of urban development studies.

The fine-grained NTL maps and hotspot diagnostics produced in this study enable several actionable use cases. (i) Sustainability monitoring: Persistent brightness hotspots can serve as real-time indicators of excessive energy consumption; the Taipei City Government could overlay our 500 m grid with its street-lighting retrofit schedule to prioritize LED upgrades in zones where VIIRS-DNB radiance remains above the 90th percentile six months after retrofitting. (ii) Land-use optimization: By comparing emerging NTL hotspots with forthcoming urban renewal parcels, planners can detect unintended spillovers of commercial activity into residential blocks and adjust zoning or floor-area-ratio incentives accordingly. (iii) Equity assessment: Identifying long-term cold spots next to underserved neighborhoods helps target public-realm lighting or nighttime transport services, advancing SDG 11 for inclusive, safe, resilient, and sustainable cities. Together, these examples illustrate how remotely sensed night‑lights can transition from descriptive analytics to an operational decision‑support tool for energy‑efficient, equitable urban development.

Nevertheless, this study had some limitations. The reliance on aggregated monthly NTL data could have obscured short-term fluctuations such as lighting changes during major events or temporary shifts in land use activities [27,51]. Additionally, the focus on a single urban area, Taipei, limits the comparative scope of the findings.

Future research to move beyond descriptive correlations, forthcoming studies should couple NTL time series with explicit policy and governance signals. In Taipei, several open‑data streams—such as district‑level urban‑renewal permits, and lighting‑efficiency ordinances released by the Department of Urban Development—provide well‑dated interventions that can be spatially georeferenced at the 500‑m grid scale.

By overlaying these policy footprints onto the VIIRS‑DNB cube, researchers can implement Bayesian difference‑in‑differences within the INLA‑SPDE framework or apply Bayesian structural time‑series models to construct counterfactual light trajectories. Such quasi-experimental designs enable the causal attribution of brightness change to specific regulations while accounting for spatial spillover and temporal autocorrelation. In addition, integrating higher‑frequency NTL composites such as daily Black Marble VNP46A2 with crowd‑sourced mobility or social‑media check‑ins could reveal short‑lived policy impacts, such as night‑market closures or energy‑curfew campaigns. These methodological extensions can help translate NTL analytics into actionable evidence for energy-efficient zoning and governance benchmarking.

## Conclusion

This study underscores the value of NTL data as a powerful tool for analyzing the spatiotemporal dynamics of urban development and socioeconomic activities. By integrating detailed land use data and utilizing a spatiotemporal Bayesian modeling framework (INLA with SPDE), we addressed three core questions. First, regarding the influence of NTL intensity, our finding reveals that commercial activities and electricity consumption are critical drivers of increased brightness, while forested and agricultural areas consistently exhibit minimal urban influence. This highlights the role of land use types in shaping NTL intensity, which can be leveraged for urban planning and environmental monitoring.

Second, by examining the spatiotemporal dynamics of NTL within the context of urban expansion and economic activity, persistent hotspots were identified in Taipei’s commercial and industrial districts. Hotspots reflect concentrated urbanization effects, whereas cold spots are associated with areas of limited development, such as forested or agricultural lands. This dynamic indicates how economic activities and urban growth patterns influence NTL distribution over time. Methodologically, this study validated the applicability of the INLA approach to a large-scale spatiotemporal NTL analysis. The INLA framework, combined with the SPDE approach, provides a scalable and efficient method for modeling complex urban dynamics [49,52]. The integration of space–time cube analysis further enhances the interpretive value by linking NTL patterns to the underlying land-use dynamics and urban processes. By translating these findings into practical strategies, this research contributes valuable tools for resilient and sustainable urban planning while setting a methodological precedent for future research.
